# Redundant Notch1 and Notch2 Signaling Is Necessary for IFNγ Secretion by T Helper 1 Cells During Infection with *Leishmania major*

**DOI:** 10.1371/journal.ppat.1002560

**Published:** 2012-03-01

**Authors:** Floriane Auderset, Steffen Schuster, Manuel Coutaz, Ute Koch, Florian Desgranges, Estelle Merck, H. Robson MacDonald, Freddy Radtke, Fabienne Tacchini-Cottier

**Affiliations:** 1 Department of Biochemistry, WHO Immunology Research and Training Center, University of Lausanne, Epalinges, Switzerland; 2 Ecole Polytechnique Fédérale de Lausanne, Swiss Experimental Cancer Research, Lausanne, Switzerland; 3 Ludwig Institute for Cancer Research Ltd, Lausanne Branch, University of Lausanne, Epalinges, Switzerland; National Institute of Health, United States of America

## Abstract

The protective immune response to intracellular parasites involves in most cases the differentiation of IFNγ-secreting CD4^+^ T helper (Th) 1 cells. Notch receptors regulate cell differentiation during development but their implication in the polarization of peripheral CD4^+^ T helper 1 cells is not well understood. Of the four Notch receptors, only Notch1 (N1) and Notch2 (N2) are expressed on activated CD4^+^ T cells. To investigate the role of Notch in Th1 cell differentiation following parasite infection, mice with T cell-specific gene ablation of N1, N2 or both (N1N2^ΔCD4Cre^) were infected with the protozoan parasite *Leishmania major*. N1N2^ΔCD4Cre^ mice, on the C57BL/6 *L. major*-resistant genetic background, developed unhealing lesions and uncontrolled parasitemia. Susceptibility correlated with impaired secretion of IFNγ by draining lymph node CD4^+^ T cells and increased secretion of the IL-5 and IL-13 Th2 cytokines. Mice with single inactivation of N1 or N2 in their T cells were resistant to infection and developed a protective Th1 immune response, showing that CD4^+^ T cell expression of N1 or N2 is redundant in driving Th1 differentiation. Furthermore, we show that Notch signaling is required for the secretion of IFNγ by Th1 cells. This effect is independent of CSL/RBP-Jκ, the major effector of Notch receptors, since *L. major*-infected mice with a RBP-Jκ deletion in their T cells were able to develop IFNγ-secreting Th1 cells, kill parasites and heal their lesions. Collectively, we demonstrate here a crucial role for RBP-Jκ-independent Notch signaling in the differentiation of a functional Th1 immune response following *L. major* infection.

## Author Summary

Infection with protozoan parasites of *Leishmania* species results in a spectrum of local or systemic diseases in humans and mammals. Overall, leishmaniasis afflicts around 12 million individuals in 88 countries worldwide. Cutaneous leishmaniasis is the most prevalent form of the disease. In order to better understand the complex molecular pathways leading to protection against the cutaneous form of the disease, we used the *Leishmania major* mouse model. Most mouse strains control *L. major* infection due to the development of a Th1 response, leading to secretion of IFNγ by T cells which promotes healing and resistance to reinfection. Notch signaling is a very conserved pathway in the regulation of cell differentiation and cell fate decision. However the contribution of Notch receptors in the response to parasite infection is not clear. In this study, we infected mice that do not express Notch1 and Notch2 receptors on the surface of their T cells. We show that these Notch receptors are key players in the development of a protective Th1 immune response against *L. major*. These results contribute to the understanding of the mechanisms involved in the development of a protective response against pathogens.

## Introduction

Following activation by pathogens, naïve CD4^+^ T cells can differentiate into several functionally distinct T helper (Th) subsets, defined by the cytokines they secrete. CD4^+^ Th1 cells secrete IFNγ as a signature cytokine and the transcription factor T-bet is essential for their differentiation. Although cytokines such as IL-12 contribute to Th1 cell differentiation, Th1 cells can be generated in the absence of cytokine signaling, demonstrating a role for other molecules in this process. Among these are Notch receptors and their ligands (Reviewed in [1], [2]). Notch signaling plays crucial roles in binary cell fate decisions in many developmental systems including the development and differentiation of immune cells. In mammals, there are four Notch receptors (Notch1-4) that are activated by five ligands (Jagged (Jag) 1, and 2, and Delta-like (Dll) 1, 3, and 4). Upon interaction with its ligand, the active intracellular domain of Notch (NICD) is released from the membrane by proteolytic cleavages and translocates into the nucleus. Once there, NICD can form a complex with recombination signal-binding protein-J (RBP-Jκ), converting it to an activator of transcription (canonical Notch signaling). Alternatively, NICD could interact with members of the NF-κB pathway (non-canonical Notch signaling) [3]. In the T cell lineage, the Notch1 receptor is essential for the development of αβ T cells [4], and Notch plays a poorly understood role in the differentiation of peripheral Th cell subsets (reviewed in [1], [5]).

The importance of Notch signaling during CD4^+^ Th1 differentiation and its correlated resolution of pathogen infection is currently unclear. Inhibitors of γ-secretase impairing Notch signaling prevented Th1 differentiation *in vitro* and *in vivo*, potentially through the blocking of T-bet expression [6]. Blocking of the Notch3 receptor using antisense N3 DNA also blocked Th1 differentiation *in vitro* [7]. In contrast, T cell-specific expression of dominant negative mastermind-like protein (MAML1), which is needed for RBP-Jκ-dependent Notch signaling, or T cell specific ablation of Notch1 or RBP-Jκ did not have an impact on Th1 differentiation *in vitro* [8] nor *in vivo* [9], [10].

The role of Notch ligands on dendritic cells instructing Th1 differentiation is also debated. Dll1 and/or Dll4 expression is upregulated *in vitro* on APCs encountering pathogens driving a CD4^+^ Th1 response [8], [11], [12]. Interaction of Notch with Dll1 promoted Th1 differentiation during *Leishmania major* infection [7]. Furthermore, Dll4 expression on DC was shown to induce Th1 cell differentiation in an IL-12-independent way [11]. On the contrary, Dll1, Jag1 and Jag2 were shown to be insufficient to instruct the differentiation of Th1 or Th2 CD4^+^ cells in absence of polarizing cytokines *in vitro*, suggesting that the induction of selective ligands by pathogens may not exert a direct influence on T helper differentiation [13], [14].

Altogether, these studies indicate a role of Notch in CD4^+^ Th1 differentiation, but it is not clear yet which member and how each member of this family contributes to this process during infection with pathogens. Most of the above studies investigated the role of Notch using total inhibition of Notch signaling, but the individual contribution and potential crosstalk of individual Notch receptors during infections with pathogens inducing CD4^+^ Th1 cells has not been investigated.

Here, mice carrying a T cell specific deletion of Notch1 (N1^ΔCD4Cre^), Notch2 (N2^ΔCD4Cre^) or both Notch1 and Notch2 (N1N2^ΔCD4Cre^) on a resistant C57BL/6 genetic background were infected with *L. major* to study the importance of Notch receptors in Th1 differentiation and resolution of the infection. We show that Notch signaling through either N1 and/or N2 induces the secretion of IFNγ by CD4^+^ Th1 cells. Moreover, using mice with T cell-specific ablation of RBP-Jκ (RBP-J^ΔCD4Cre^), we show that Th1 differentiation is induced mainly by non-canonical (RBP-Jκ-independent) Notch signaling. Collectively, our data indicate that Notch signaling drives the differentiation of *L. major*-specific IFNγ-secreting Th1 cells required to mount an efficient immune response against this parasite.

## Results

### Notch affects the development of a protective *L. major*-specific Th1 cell response

**Figure 1:**
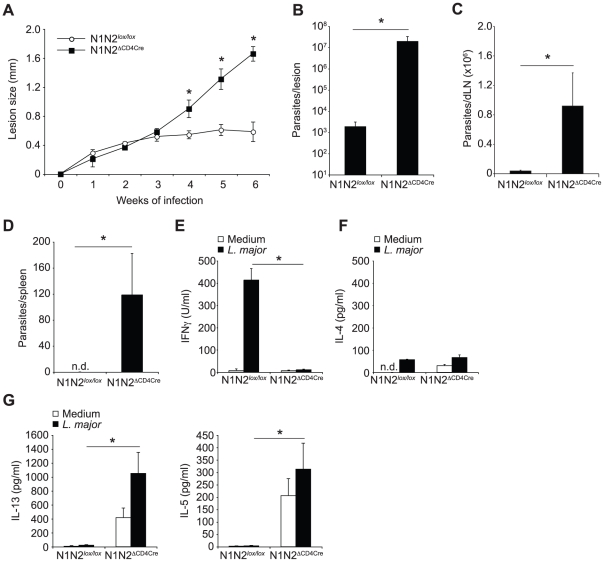
N1N2^ΔCD4Cre^ mice on the C57BL/6 *L. major* resistant background are susceptible to infection. (A) N1N2^ΔCD4Cre^ and control N1N2*^lox/lox^* mice were infected with 3×10^6^ *L. major* promastigotes and lesion size measured weekly. Dots represent group mean of lesion size ± SEM. (B, C) Six weeks after infection, parasite load was assessed by LDA in footpads (B), dLN (C) and spleen (D). Histograms represent the mean number of parasite ± SEM (n≥3 mice per group). (E–G) IFNγ (E), IL-4 (F), IL-13 and IL-5 (G) secretion was quantified in supernatants of draining lymph node cells restimulated or not with UV-irradiated *L. major* 6 weeks after infection. Mean cytokine secretion ± SEM are given (n≥3 mice per group). Data are representative of at least 3 individual experiments. n.d. not-detectable. * p-value<0.05 versus control mice.

To investigate Notch function in Th1 cell development we infected mice with *L. major*, a parasite promoting a predominant Th1 immune response in most strains of mice including C57BL/6 [15]. Of the four Notch receptors, only N1 and N2 are expressed in activated T cells [8], [16]. Thus, to investigate the effect of T cell ablation of these two receptors (N1N2^ΔCD4Cre^) on CD4^+^ Th1 differentiation and the consequent resolution of *L. major* infection, N1N2^ΔCD4Cre^ and control N1N2*^lox/lox^* mice on the *L. major* resistant C57BL/6 genetic background were inoculated with the parasite. In contrast to N1N2*^lox/lox^* control mice that developed a small self-healing lesion, N1N2^ΔCD4Cre^ were unable to heal their lesions (Figure 1A). In addition, *L. major*-infected N1N2^ΔCD4Cre^ mice failed to control parasite load at the site of parasite inoculation (Figure 1B) and *L. major* disseminated to the lymph nodes and spleen (Figure 1C, D).

The impact of the absence of Notch on T cells in the differentiation of CD4^+^ IFNγ-secreting Th1 cells was assessed six weeks after infection. N1N2^ΔCD4Cre^ and control draining lymph node (dLN) cells were restimulated *in vitro* with UV-irradiated *L. major* and cytokine levels measured. Strikingly, secretion of IFNγ was abrogated in supernatants of N1N2^ΔCD4Cre^ dLN cells, while high levels of this cytokine were measured in dLN from infected control mice (Figure 1E). Similarly low IL-4 levels were measured in each group (Figure 1F). IL-13 and IL-5 were found predominantly in N1N2^ΔCD4Cre^ dLN cells (Figure 1G). The persistence of parasites in the dLN of *L. major* infected N1N2^ΔCD4Cre^ but not N1N2*^lox/lox^* mice was sufficient to induce IL-13 and IL-5 secretion by T cells (Figure 1G), albeit at a lower level than that reached following stimulation with exogenous addition of *L. major*. These results show that Notch signaling contributes to the generation of IFNγ-secreting CD4^+^ T cells, which are essential in the control of parasite load and lesion size. The absence of Notch expression on T cells, while preventing IFNγ secretion, favored the development of IL-13- and IL-5-secreting cells.

### N1 or N2 expression on T cells is sufficient to drive Th1 cell differentiation

**Figure 2:**
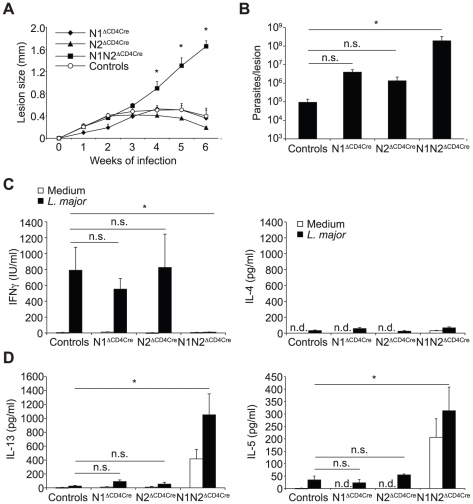
N1 and N2 alone can drive CD4^+^ Th1 differentiation. (A) N1^ΔCD4Cre^, N2^ΔCD4Cre^, N1N2^ΔCD4Cre^, and control mice were infected with 3×10^6^ *L. major* promastigotes and lesion size measured weekly. Data are represented as the mean of lesion size ± SEM with n≥3 mice per group. (B) Parasite load in the lesion was assessed by LDA 6 weeks after infection. Mean parasite number is given ± SEM (n≥3 mice per group) (C, D) Six weeks after infection, IFNγ (C), IL-4 and IL-13 (D) secretion was assessed in supernatant of dLN cells restimulated or not with UV-irradiated *L. major* for 72 h. Histograms show the mean cytokine secretion ± SEM (n≥3 mice per group). n.d. not-detectable, n.s. not significant. * p-value<0.05 versus control mice.

N1^ΔCD4Cre^ mice are able to develop a protective Th1 response in response to *L. major* inoculation [9]. The inability of *L. major*-infected N1N2^ΔCD4Cre^ mice to develop a protective Th1 immune response suggested that N2 could be the receptor involved in Th1 differentiation. To investigate this, N2^ΔCD4Cre^ and N2*^lox/lox^* control mice were infected with *L. major* and evolution of lesion size and development of immune response were compared to that developing in N1^ΔCD4Cre^, and N1N2^ΔCD4Cre^ infected mice. N2^ΔCD4Cre^ mice were able to control their lesion size (Figure 2A) and parasitemia (Figure 2B) as well as N1^ΔCD4Cre^ and control mice, unlike N1N2^ΔCD4Cre^ mice. To analyze their immune response, cytokine secretion by dLN cells was analyzed. *L. major*-infected N2^ΔCD4Cre^ dLN cells secreted similar levels of IFNγ than *L. major*-infected N1^ΔCD4Cre^ and control mice. Low levels of IL-4, IL-5 and IL-13 were similarly measured in their dLN cells (Figure 2C).

**Figure 3:**
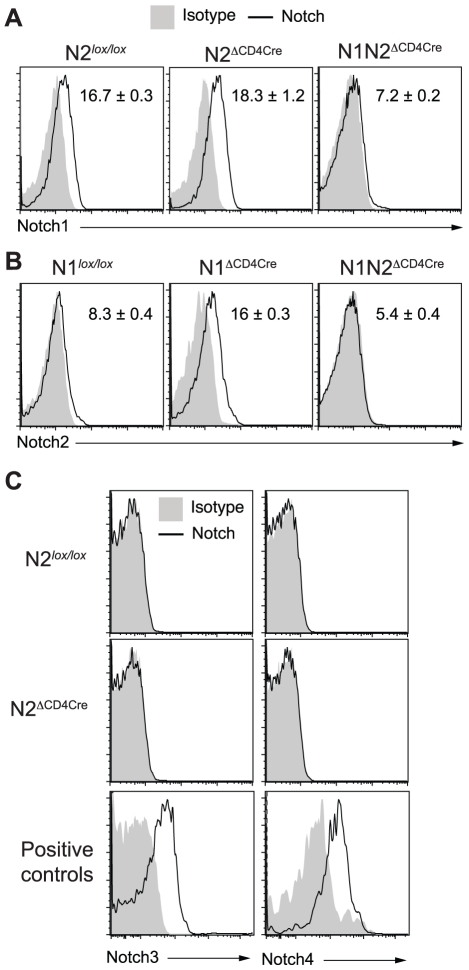
Increased N2 expression can compensate the absence of N1 on *L. major* stimulated CD4^+^ T cells. (A–C) Three weeks after *L. major* infection, dLN cells from the indicated mouse strains were isolated and restimulated for 16 h with UV-treated *L. major*. Notch1 (A), Notch2 (B), Notch3 and Notch4 (C) expression by CD4^+^ T cells was assessed by FACS. CD11c^+^CD8α^+^ splenic dendritic cells and CD4^−^CD8^−^CD25^+^ thymocytes were stained as positive controls for Notch3 and Notch4 respectively. Representative flow cytometry plots are shown. Numbers in plots represent mean fluorescence intensity MFI ± SEM of ≥3 mice per group. Data are representative of 3 independent experiments.

N1N2^ΔCD4Cre^ mice are susceptible to *L. major* infection and fail to induce IFNγ secretion by CD4^+^ T cells, indicating that expression of either N1 or N2 is sufficient to induce CD4^+^ Th1 differentiation in N2^ΔCD4Cre^ or N1^ΔCD4cre^ mice, respectively. To investigate if following parasite inoculation, compensatory expression of one or all of the Notch receptors on T cells could occur, dLN cells of *L. major* infected N1^ΔCD4Cre^ or N2^ΔCD4Cre^ mice were stimulated *in vitro* with *L. major*, and Notch expression on their CD4^+^ T cells measured by FACS. N1 expression was significantly and similarly expressed in both N2^ΔCD4Cre^ and N2*^lox/lox^* CD4^+^ T cells (Figure 3A). Low levels of N2 surface expression were induced following restimulation of control dLN T cells with *L. major*, however, a significantly higher induction of N2 was measured in *L. major*-activated N1^ΔCD4cre^ CD4^+^ T cells (Figure 3B), suggesting that compensatory mechanisms allow increased N2 expression in absence of N1. No expression of N3 and N4 mRNA or proteins was detectable on T cells of all genotypes, in contrast to positive control cells (Figure 3C and data not shown). Altogether, these data reveal that signaling through either N1 or N2 is sufficient for the generation of functional Th1 cells following infection with *L. major*, and that in absence of N1 compensatory higher expression of N2 is measured on T cells.

### Susceptibility to *L. major* infection in absence of Notch on T cells results mainly from lack of IFNγ secretion

**Figure 4:**
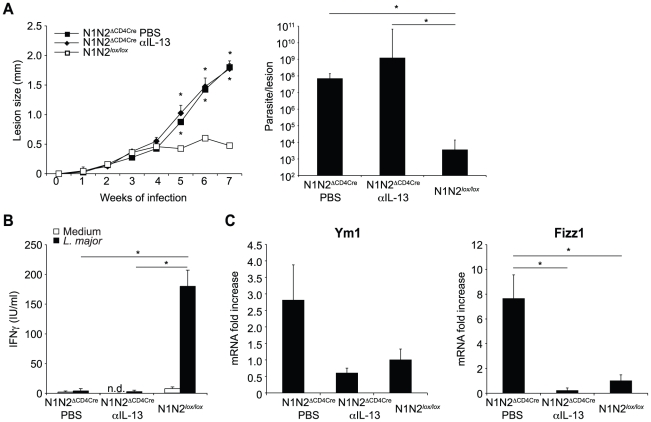
Treatment with anti-IL-13 does not restore resistance of N1N2^ΔCD4Cre^ mice to *L. major*. (A) N1N2^ΔCD4Cre^ and control N1N2*^lox/lox^* mice were infected s.c. with 3×10^6^ *L. major* promastigotes. At day 21 of infection, N1N2^ΔCD4Cre^ mice were treated i.p. with either anti-IL-13 mAb or PBS as control. Treatment was repeated once a week until the end of the experiment, and lesion development was monitored. Group means of lesion size ± SEM (n≥3 mice per group) are represented. The parasite load at the site of infection was assessed by LDA 47 days post infection. Group means of parasite number are given ± SEM (n≥3 mice per group). (B) CD4^+^ T cells were isolated by MACS from dLN of *L. major*-infected mice 47 days post infection and restimulated with or without UV-treated *L. major* in presence of irradiated syngenic splenocytes. IFNγ level was measured in supernatant after 72 h of stimulation. Data are expressed as the group mean ± SEM of cytokine measurement of n≥3 draining lymph nodes (C) Ym1 and Fizz1 mRNA expression was analyzed in dLN cells by quantitative real-time PCR and normalized to HPRT mRNA expression. Results are represented as fold-increase in mRNA levels relative to levels measured in control mice ± SEM (n≥3 mice per group). Data are representative of three independent experiments. Similar results were obtained when anti-IL-13 was administrated 7 days post infection (data not shown). * p-value<0.05 versus control mice.

Draining LN CD4^+^ T cells of *L. major*-infected N1N2^ΔCD4Cre^ mice fail to secrete IFNγ but release high levels of IL-13. This cytokine has been associated with susceptibility to *L. major* infection, mostly by preventing the classical activation of macrophages by IFNγ [17]. To investigate if susceptibility of N1N2^ΔCD4Cre^ mice resulted from a lack of IFNγ secretion and/or from the presence of high levels of IL-13, IL-13 was neutralized with an anti-IL-13 mAb after inoculation of *L. major* in N1N2^ΔCD4Cre^ and N1N2*^lox/lox^* control mice. No effect was observed in lesion development and parasite control in IL-13-depleted N1N2^ΔCD4Cre^ mice, that developed unhealing lesions similar to mice treated with PBS (Figure 4A). Similar low levels of IFNγ were measured in isolated CD4^+^ T cells of mice depleted or not of IL-13 (Figure 4B). The efficacy of the anti-IL-13 treatment was confirmed by measuring dLN levels of Fizz1 and Ym1 expression, two markers of alternative macrophage activation. The mRNA levels of both markers were decreased in dLN of anti-IL-13-treated N1N2^ΔCD4Cre^ mice (Figure 4C). Collectively, these data demonstrate that the non-healing phenotype measured in N1N2^ΔCD4Cre^ mice results primarily from the decreased IFNγ secretion by CD4^+^ T cells which does not allow activation of macrophage to kill the intracellular parasites. The high levels of IL-13 which induce alternative macrophage activation do not play a critical role in the failure of macrophages to kill the parasites, as in absence of IFNγ, macrophages are already not classically activated.

### Notch signaling prevents the release but not the transcription of IFNγ by CD4^+^ dLN cells

**Figure 5:**
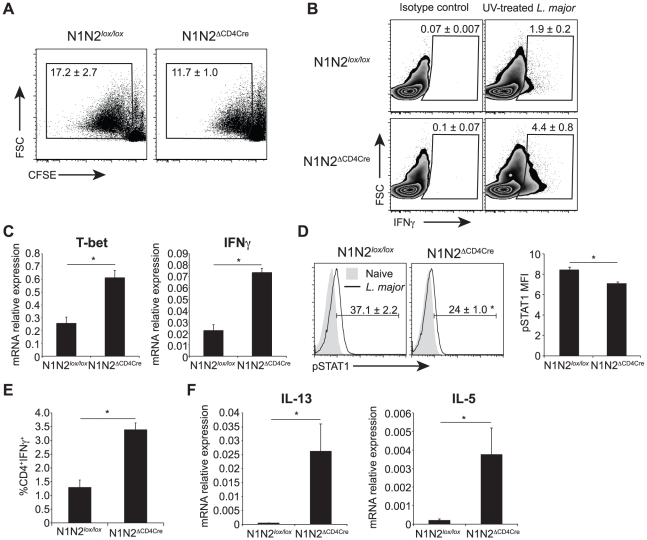
N1N2^ΔCD4Cre^ mice transcribe T-bet and IFNγ in dLN CD4^+^ T cells but do not secrete it. (A) Proliferation of CD4^+^ T cells was assessed by FACS. Draining LN cells of *L. major*-infected mice were isolated 6 weeks after infection, stained with CFSE and restimulated with UV-treated *L. major* for 72 h. Representative flow cytometry plots gated on CD4^+^ T cells are shown. Numbers in plots represent the mean percentage of proliferating cells ± SEM for 5 mice. (B) Intracellular levels of IFNγ were analysed by FACS in *L. major*-infected dLN cells restimulated for 72 h with UV-treated *L. major*. Representative flow cytometry plots are given. Numbers in plots represent the mean percentage of IFNγ^+^ cells within CD4^+^ T cells ± SEM for 5 mice. (C) Draining LN CD4^+^ T cells from N1N2^ΔCD4Cre^ and N1N2*^lox/lox^* mice were sorted by FACS 21 days post *L. major* infection, T-bet and IFNγ mRNA levels were analyzed by quantitative RT-PCR. Data are represented as the mean ± SEM mRNA transcript levels normalized to HPRT mRNA levels (n≥3 mice per group). (D) Phosphorylation of STAT1 was assessed by FACS on dLN cells of N1N2^ΔCD4Cre^ and N1N2*^lox/lox^* mice 3 weeks post infection. Naive mice were used as control. Representative flow cytometry plots gated on CD4^+^ T cells are shown. Numbers in quadrants indicate the mean frequency of pSTAT1^+^CD4^+^ T cells ± SEM. pSTAT1 mean fluorescence intensity MFI ± SEM is shown (n≥3 mice per group). (E) Draining LN cells of *L. major*-infected mice were restimulated *ex vivo* with PMA/ionomcyin for 4 h and level of intracellular IFNγ was assessed by FACS. The frequency of CD4^+^IFNγ^+^ T cells is given ± SEM for n≥3 mice per group. (F) mRNA expression of IL-13 and IL-5 was analyzed by quantitative real-time PCR in dLN cells isolated from N1N2^ΔCD4Cre^ and N1N2*^lox/lox^* mice 6 weeks post *L. major* infection. Results are given as mean mRNA expression relative to HPRT ± SEM for n≥3 mice per group. Data are representative of 2–3 individual experiments. * p-value<0.05 versus control mice.

We then investigated if the impaired IFNγ secretion measured in N1N2^ΔCD4Cre^ CD4^+^ T cells could result from defective *in vitro* proliferation. To this end, dLN cells of *L. major*-infected N1N2^ΔCD4Cre^ and control mice were stained with CFSE and restimulated for 72 h with *L. major*. CD4^+^ T cells of both N1N2^ΔCD4Cre^ and control proliferated in response to the parasite. N1N2^ΔCD4Cre^ showed a slightly lower CD4^+^ T cell proliferation compared to that of N1N2*^lox/lox^* CD4^+^ T cells but the difference was not statistically significant (Figure 5A). Despite comparable proliferation, IFNγ was not secreted in response to *L. major* stimulation. However, high levels of intracellular IFNγ were measured by FACS in N1N2^ΔCD4Cre^ CD4^+^ T cells restimulated with *L. major* for 72 h, in absence of PMA-ionomycin stimulation (Figure 5B). To further determine at which level the absence of N1 and N2 on T cells affects secretion of IFNγ in the dLN of *L. major*-infected mice, mRNA levels of IFNγ and T-bet, the major transcription factor of Th1 cells, were measured *ex vivo* on FACS sorted CD4^+^ T cells 3 weeks after infection. Reduced levels of secreted IFNγ did not result from impaired transcription of T-bet or IFNγ mRNA as demonstrated by higher levels of both T-bet and IFNγ mRNA measured in N1N2^ΔCD4Cre^ CD4^+^ T cells compared to those measured in CD4^+^ T cells of control mice (Figure 5C). No defect in activation status or in CD4^+^ T cell number was measured in dLN cell N1N2^ΔCD4Cre^ mice (Figure S1). IFNγ signaling is mediated by STAT1 phosphorylation and IFNγ was reported to signal to the majority of cells throughout the dLN during a Th1 response after *T. gondii* infection [18]. To further show that secretion of IFNγ is impaired in CD4^+^ T cells during infection, we measured STAT1 phosphorylation in dLN CD4^+^ T cells of *L. major*-infected N1N2^ΔCD4Cre^ mice. STAT1 phosphorylation was detected in CD4^+^ T cells of control mice while only minimal STAT1 phosphorylation was measured in CD4^+^ T cells of N1N2^ΔCD4Cre^ dLN cells (Figure 5D). These data confirm that in absence of N1 and N2 on T cells, IFNγ secretion by CD4^+^ T cells is impaired, thereby preventing IFNγ-induced STAT1 phosphorylation occurring *in vivo*. The impairment of IFNγ secretion is antigen-specific and not due to an intrinsic secretion default in N1N2^ΔCD4Cre^ mice as revealed by the high levels of IFNγ detected by intracellular staining following TCR-independent T cell stimulation (PMA-ionomycin) *ex vivo* (Figure 5E). In addition, defective secretion could be overcome *in vitro* by antibody-mediated CD3 crosslinking stimulation (Figure S2A).

In the same line, naïve N1N2^ΔCD4Cre^ CD4^+^ T cells stimulated *in vitro* in the presence of standard Th1 polarizing conditions followed by stimulation with anti-CD3/CD28 for 48 hours were able to develop into IFNγ-secreting cells (Figure S2B). In contrast, the strong increase in IL-13 and IL-5 mRNA levels measured in dLN of *L. major*-infected mice (Figure 5F) correlated with the high secretion levels of these cytokines in *L. major*-stimulated CD4^+^ T cells. Altogether, these data reveal that following inoculation of *L. major*, absence of N1 and N2 on T cells prevents the release of IFNγ by CD4^+^ T cells, favoring the differentiation of IL-13- and IL-5-secreting cells. This effect is obscured *in vitro* by exogenous addition of high amounts of cytokines and/or antigen-non specific activation of T cells.

### Notch signaling driving CD4^+^ Th1 differentiation occurs in absence of the RBP-Jκ transcription factor

Mice with dominant negative MAML (DNMAML) protein preventing the canonical transcriptional activation by all four Notch receptors were previously reported to be able to control infection with *L. major* and to have normal levels of IFNγ in their dLN CD4^+^ T cells [10]. Different strains of *L. major* may induce distinct type of T helper immune response [19]. To further insure that the different outcomes on the differentiation of Th1 cells measured in theirs (*L. major* Friedlin) and the present studies (*L. major* LV39) were not due to differences in the *L. major* strains used, N1N2^ΔCD4Cre^ mice were infected with two other *L. major* strains (Friedlin or IR175). N1N2^ΔCD4Cre^ mice infected with these two *L. major* strains failed to develop an efficient Th1 response with decreased secretion of IFNγ and increased secretion of IL-13 and IL-5 by their dLN T cells and high intralesional parasite load (Figure S3). These data show that N1 and N2 are required for Th1 differentiation following infection with different strains of *L. major*.

**Figure 6:**
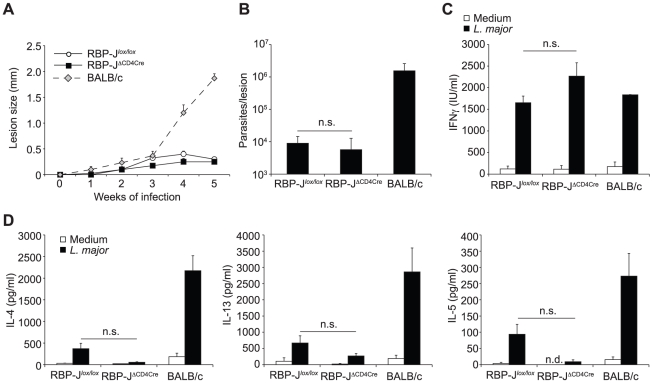
The impact of Notch signaling on Th1 differentiation is RBP-Jκ-independent. (A) RBP-Jκ^ΔCD4Cre^ and RBP-Jκ*^lox/lox^* mice were infected s.c. with 3×10^6^ *L. major* and lesion size measured weekly. Group mean of lesion size ± SEM for n≥3 mice per group is shown. (B) Parasite load in footpad was analyzed by LDA 5 weeks post infection. Data represent mean parasite number ± SEM for n≥3 mice per group. (C, D) IFNγ (C), IL-4, IL-13 and IL-5 (D) levels were measured in supernatant of dLN cells isolated from *L. major*-infected mice 5 weeks post infection and restimulated for 72 h with or without UV-treated *L. major*. Group mean of cytokine secretion ± SEM is given (n≥3 mice per group). n.s. not significant. * p-value<0.05 versus control mice.

Having ruled out a potential effect due to the strain of *Leishmania* used, the lack of effect of DNMAML on Th1 differentiation [10] suggested that Notch signaling may not drive Th1 cell differentiation through the NICD–MAML–RBP-Jκ transcriptional activation complex. To investigate if the requirement of Notch signaling for CD4^+^ Th1 differentiation and the associated resolution of the lesion could be RBP-Jκ-independent, we infected RBP-Jκ^ΔCD4Cre^ and RBP-Jκ*^lox/lox^* control mice with *L. major*. No difference in lesion development (Figure 6A) nor parasite control was measured between RBP-Jκ^ΔCD4Cre^ and control mice (Figure 6B). Furthermore, the development of CD4^+^ IFNγ-secreting Th1 cells was normal, as revealed by high levels of IFNγ secretion by dLN T cells, and low levels of IL-13 and IL-5 (Figure 6C). These results demonstrate that RBP-Jκ-independent Notch signaling is required for CD4^+^ Th1 differentiation following *L. major* infection.

## Discussion

The development of Th1 cells, through their secretion of IFNγ, contributes to a number of protective effects against many pathogens. Despite a growing understanding on the mechanisms leading to T helper differentiation these last years (reviewed in [20]), there are still unresolved issues including the identification of which receptor triggering leads to Th1 differentiation. The role of Notch in Th1 differentiation has been controversial, depending on the mode of activation/deactivation of Notch [1], [5]. Our data showing the crucial role of RBP-jκ-independent Notch signaling in the differentiation of IFNγ-secreting T cells help to reconcile discrepant results obtained using different loss or gain of function approaches that attributed or not a role for Notch signaling in Th1 differentiation.

Here, using mice with ablation of Notch in their T cells, we demonstrate that expression of either N1 or N2 on T cells is necessary and sufficient for the differentiation of IFNγ-secreting Th1 cells and the consequent control of infection. N1N2^ΔCD4Cre^ mice infected with *L. major* failed to mount a protective Th1 immune response while mice with single deletion of N1 (this study and [9]) or N2 in their T cells developed a protective Th1 immune response. In control mice, N1 is the only receptor expressed at significant levels at the surface of anti-CD3 and *L. major*-activated CD4^+^ T cells. There appears to be functional redundancy of N1 and N2 in driving CD4^+^ T helper 1 differentiation. Expression of N2 is low in activated T cells of *L. major*-infected control mice, but compensatory increased N2 expression was measured in absence of N1 expression. This suggests that N1 is the main receptor involved in signaling leading to the induction of IFNγ secretion by T cells following infection with *L. major*, but that in absence of N1, higher levels of N2 can compensate its absence. Functional redundancy of N1 and N2 was previously suggested in N1IAS mice that had decreased but not abrogated IFNγ secretion [6], however the expression of Notch receptors was not assessed in that study. Of note, we show here that N2 is the only receptor that could functionally substitute for N1 in driving Th1 differentiation *in vivo*, and T cell expression of N3 or N4 were not detected in presence or absence of N1 and/or N2 in CD4^+^ T cells of *L. major*-infected mice. In addition, N1N2^ΔCD4Cre^ mice do not control infection revealing that N3 and N4 are not functionally redundant in driving IFNγ secretion by CD4^+^ T cells. Overexpression of N3 intracellular domain (N3IC) in T cells was previously reported to induce IFNγ secretion *in vitro* following anti-CD3 activation, while overexpression of N1IC did not, suggesting that N3 could be involved in Th1 differentiation [7]. Together with our reported increased expression of N2 in absence of N1, these results show that individual Notch receptors may potentially drive IFNγ secretion by CD4^+^ T cells, but during *L. major* infection N1, and to a lesser extent N2 appear to be the only receptors involved in driving Th1 differentiation.

It was reported that N1 could regulate Th1 cell differentiation by interacting with CSL sequences present in the promoter of the *Tbx21* gene which codes for T-bet, the master regulator of Th1 cell differentiation [6]. However, in another study, Notch was not found to reside at the *Tbx21* promoter [21]. In addition, we show here that mice with specific ablation of RBP-Jκ in their T cells, unlike N1N2^ΔCD4Cre^ mice, are able to mount a Th1 response and heal their lesion following infection with *L. major*. These results show that the Notch signaling playing a major role in the differentiation IFNγ-secreting cells following infection with *L. major* occurs in absence of a CSL/RBP-Jκ-transcription complex. In line with these data, it was previously reported that mice that conditionally expressed a dominant negative MAML protein (DNMAML) and thereby are deprived of RBP-Jκ-mediated transcriptional activation of all Notch receptors, were able to develop a protective Th1 immune response following *L. major* infection [10]. The present results show that Notch receptors are crucial to trigger secretion of IFNγ by CD4^+^ T cells in a CSL/RBP-Jκ-independent manner.

The nature of a CSL/RBP-Jκ-independent Notch pathway is complex and not yet defined [22]. It has been reported that Notch can associate with the nuclear factor κB (NF-κB) proteins p50 and p65. Furthermore, Notch1-NF-κB complexes could be immunoprecipitated from the *Ifng* promoter despite the lack of consensus binding sites for RBP-Jκ in the promoter of this gene [23]. This suggested that N1ICD could contribute to Th1 differentiation in a RBP-Jκ-independent way leading to the hypothesis of a connection between Notch, NF-κB and Th1 differentiation [1], [5]. Of note, NF-κB p50 is required for optimal Th1 development and *L. major*-infected NF-κB1 knockout mice show a susceptible phenotype associated with defective secretion of IFNγ [24]. However in that study, failure to secrete IFNγ was linked to a major defect in CD4^+^ T cell proliferation measured *in vitro*, while we did not detect such impairment of CD4^+^ T cell proliferation in Notch deficient CD4^+^ T cells. Thus Notch may interact with distinct transcription factors involved in the secretion of IFNγ by Th1 cells and further molecular studies will be needed to determine the nature of these factors as well as the molecular mechanisms involved in the RBP-Jκ-independent Notch signaling during Th1 differentiation.

Notch signaling is required for proper secretion of IFNγ by CD4^+^ Th1 cells following antigen-specific stimulation. Interestingly, increased expression of T-bet and IFNγ mRNA was measured in dLN CD4^+^ T cells of *L. major*-infected N1N2^ΔCD4Cre^ mice revealing that Notch signaling does not prevent the differentiation of “competent” CD4^+^ Th1 cells [25], but appears to act downstream of it. The increase in T-bet and IFNγ mRNA measured in CD4^+^ N1N2^ΔCD4Cre^ T cells suggests that intact Notch signaling regulates the extent transcription for these genes *in vivo*. Low levels of STAT1 phosphorylation in dLN CD4^+^ T cells confirmed that only very small amounts of IFNγ protein, maybe released by NK cells, are present in the dLN of *L. major*-infected N1N2^ΔCD4Cre^ mice. In absence of IFNγ, mice on the resistant C57BL/6 genetic background develop a Th2 immune response, with high levels of IL-4, IL-5 and IL-13 cytokines [26]. Accordingly, impaired secretion of IFNγ by CD4^+^ T cells of *L. major*-infected N1N2^ΔCD4Cre^ mice allowed the differentiation of IL-5- and IL-13-secreting Th2 cells. However, no increased secretion of IL-4 was measured in CD4^+^ T cells of N1N2^ΔCD4Cre^ *L. major*-infected mice, in line with the previously reported crucial importance of Notch in driving IL-4 secretion by CD4^+^ T cells [8], [21], [27]. Interestingly, absence of Notch did not impair the differentiation of other Th2 effector T cells, suggesting that following *L. major* infection, Notch is acting directly on the IL-4 promoter, as previously reported [8], and does not affect the differentiation of IL-13- and IL-5-Th2 secreting cells.

Notch signaling is resulting from an interaction between Notch receptors and ligands on antigen presenting cells. Several ligands have been linked to Th1 differentiation in distinct experimental models of disease and Delta-like ligands have been linked to Th1 differentiation or impaired Th2 differentiation [8], [11], [12], [28], [29]. Dll1 stimulation was shown to trigger Th1 development following *L. major* infection, but it was not determined which Notch receptor was interacting with this ligand [7]. The present study shows that either N1 or N2 could be interacting with Dll1. Whether other Notch ligands are involved in Notch signaling during *Leishmania* infection remains to be investigated. Interestingly, it was reported recently that within the 6q27 gene cluster, the Dll1 gene was linked to susceptibility to visceral leishmaniasis, and reduced Dll1 expression was measured in VL patients in Sudan, Brazil, and Northen India [30]. Thus genetic regulation of one of the Notch ligand, such as the downregulation of Dll1 expression appears to have major consequences on susceptibility to VL. Together with the present study, it reveals that a proper regulation of the Notch signaling pathway during infection with *Leishmania* parasites is essential for the development of a protective response against these parasites.

Further understanding of the mechanisms by which Notch receptors regulate the differentiation of IFNγ-secreting Th1 cells as well as the ligands involved in this process should contribute to the development of new vaccines and immunotherapeutic targets towards *Leishmania* pathology, as well as in other infections requiring protective IFNγ-secreting CD4^+^ Th1 immune response.

## Materials and Methods

### Ethics statement

This study was carried out in strict accordance with the recommendations in the Guide for the care and use of laboratory animals from the Department of security and Environment (DSE) from the state of Vaud, Switzerland. The protocol has been approved by the Ethics and Veterinary office regulations of the state of Vaud (SAV), Switzerland. Our laboratory has the administrative authorization numbers 1266-3, -4 and -5.

### Mice

The following T cell specific gene-targeted mice were generated by crossing floxed Notch1 [31], floxed Notch2 [32], double floxed Notch1-Notch2 or floxed RBP-Jκ [33] mice, with mice carrying the CD4Cre transgene [34]. N1*^lox/lox^*, N2*^lox/lox^*, N1N2*^lox/lox^* and RBP-Jκ*^lox/lox^* littermates were used as controls. All mice were on a C57BL/6 genetic background. T cell-specific deletion of Notch and RBP-Jκ was verified for each strain by PCR. All mice were bred and maintained under pathogen-free conditions in the animal facility at the CIIL, Epalinges, Switzerland.

### Parasites and infections

*Leishmania major* LV39 (MRHO/Sv/59/P strain) was used. Mice were infected s.c. with 3×10^6^ stationary phase *L. major* promastigotes in the footpad. Parasite load was assessed by limiting dilution analysis (LDA). Treatment with CNTO 134, a rat anti-mouse IL-13 mAb [35], a gift from Centocor, Inc, was initiated either six days or 21 days after infection, with injection of 500 µg i.p., once weekly in N1N2^ΔCD4Cre^ mice, while a control group similarly infected was treated with control IgG or PBS. As no biological differences were observed between *L. major*-infected mice treated with control IgG or PBS, PBS was used as control vehicle for CNTO 134.

### Lymphocyte culture and cytokine assays

Draining lymph node cells were cultured ± UV-irradiated *L. major* promastigotes or anti-CD3 (clone 145-2C11, eBioscience) for 72 h. CD4^+^ T cells were isolated by MACS (Miltenyi Biotec), and cultured in the presence of irradiated C57BL/6 splenocytes. For *in vitro* experiment, naïve CD4^+^CD62L^+^ T cells were isolated by MACS and cultured as previously described [9]. The cytokine content of the cell supernatant was measured by ELISAs. IFNγ with a limit of detection of 10 IU/ml. IL-4, IL-5 (OptEIA from BD Biosciences) and IL-13 (DuoSet from R&D Systems) cytokines were analyzed with commercial kits.

### mRNA extraction and Real-Time PCR

Extraction of total RNA was performed as previously described [36]. Quantitative Real-Time PCRs were done using SYBR Green and a LightCycler system (Roche). Each cytokine mRNA was normalized to the relative hypoxanthine phosphoribosyltransferase (HPRT) endogenous mRNA expression, and represented as arbitrary units as described previously [36]. Primers used were previously described [36], [37], [38].

### Flow cytometry

Draining lymph node cells were isolated 3 weeks after *L. major* infection. Phosphorylation of STAT1 at tyrosine 701 (pY701) was detected by intracellular staining using an Alexa Fluor 488 conjugated anti-Stat1, PhosFlow Fix Buffer I and Perm Buffer III (BD Biosciences) according to manufacturer's instructions. CD4-PE-Cy5 and CD44-APC (eBiosciences) were used to stain cell surface. To assess Notch receptor expression, dLN cells were isolated and restimulated with UV-irradiated *L. major* for 16 hours. Cells were stained with anti-N1, anti-N2 biotinylated mAbs [16], followed by Streptavidin-PE, -APC (eBiosciences), PE-conjugated anti-N3 and anti-N4 (Biolegends). CD4^−^CD8^−^CD25^+^ thymocytes were used as positive control for N3 staining, and splenic CD8α^+^CD11c^+^ dendritic cells were used as positive control for N4 staining. CD4^+^ T cells were gated using TCRβ-APC and CD4-FITC (eBiosciences) mAbs. Dead cells were excluded using 7AAD (BD Pharmingen). For T cell proliferation, dLN cells were isolated 6 weeks post *L. major* infection and stained with CFSE (Molecular Probes). Cells were then restimulated ± UV-irradiated *L. major* promastigotes for 72 h and analyzed by FACS. The following monoclonal Ab conjugates were used: CD4-PE-Cy5, CD8-APC, B220-Pe-TexasRed (eBioscience) and dead cells were excluded with DAPI. Intracellular IFNγ was analyzed in dLN cells isolated 6 weeks post infection and restimulated with PMA (50 ng/ml), ionomycin (500 ng/ml) and BrefelinA (1 µg/ml) for 4 h. Cells were stained for surface marker with the following mAb conjugates: CD4-PE-Cy5, CD8-APC (eBiosciences). Intracellular IFNγ-PE was detected with an anti-IFNγ-PE (BD Pharmingen). All analyses were performed on a FACS Calibur or a LSR II (Becton Dickinson) flow cytometers and data processed with FlowJo (TreeStar).

### Statistical analysis

Data were analyzed using the Student's t-test for unpaired data.

## Supporting Information

Figure S1 **CD4^+^ T cell activation is not affected by absence of Notch receptors.** (A) Draining LN cells of N1N2^ΔCD4Cre^ and N1N2*^lox/lox^* mice were isolated 6 weeks post *L. major* infection. Total number of cells, frequency and number of CD4^+^ T cells within dLNs are given ± SEM (n≥3 mice per group). (B) CD44 and CD62L expression was assessed by FACS on CD4^+^ T cells 6 weeks post infection. Representative FACS plots are shown. Histograms and numbers in quadrants represent the mean frequency of respective cells within CD4^+^ T cells ± SEM. n.s. not significant. * p-value<0.05 versus control mice. This is a representative experiment of three.
(EPS)

Figure S2 **Strong antigen-independent stimulation of CD4^+^ N1N2^ΔCD4Cre^ cells can promote their IFNγ release** ***in vitro*** **.** (A) Draining LN cells of N1N2^ΔCD4Cre^ and N1N2*^lox/lox^* mice were isolated 6 weeks post *L. major* infection and restimulated with anti-CD3 for 72 h and the IFNγ secretion was analyzed in supernatants by ELISA. The mean IFNγ secretion ± SEM is given for n≥3 mice per group. (B) Naïve CD4^+^CD62L^+^ T cells were isolated by MACS and differentiated *in vitro* under unbiased, Th1 or Th2 condition. IFNγ level was measured in supernatant by ELISA. A representative experiment of five is shown. n.d. not detectable. * p-value<0.05 versus control mice.
(EPS)

Figure S3 **Impaired Th1 cell development in N1N2^ΔCD4Cre^ mice is not dependent on the** ***L. major*** **parasite strain.** N1N2^ΔCD4Cre^ and control N1N2*^lox/lox^* mice were infected s.c. with 3×10^6^ *L. major* promastigotes of *L. major* Friedlin (A) or IR75 (B) and LV39 (C) strains. 6 weeks after infection, CD4^+^ T cells were isolated, restimulated or not with UV-irradiated *L. major* Friedlin (A), IR75 (B) or LV39 (C) in presence of irradiated spleen cells, and 72 hours later, IFNγ and IL-13 secretion was quantified in supernatants. Mean cytokine secretion ± SEM are given (n≥3 mice per group). (D) The intralesional parasite load was assessed by LDA 6 weeks post infection with *L. major* Friedlin and IR75. Mean number of parasite per lesion is represented ± SEM for n≥3 mice per group. Data are representative of at least 3 individual experiments. * p-value<0.05 versus control mice.
(EPS)
